# Real-World Treatment Patterns of Metastatic Epidermal Growth Factor Receptor (EGFR)-Mutated Non-small Cell Lung Cancer Patients From the Integra Connect Database

**DOI:** 10.7759/cureus.97347

**Published:** 2025-11-20

**Authors:** Tao Ran, Iris Lin, Cindy Chen, Shawn S Du

**Affiliations:** 1 Oncology, Real World Value and Evidence, Johnson and Johnson, Titusville, USA; 2 Internal Medicine, Real World Value and Evidence, Johnson and Johnson, Titusville, USA

**Keywords:** egfr mutations, immunotherapy, metastatic non-small cell lung cancer, real-world evidence, treatment patterns

## Abstract

Introduction

Targeted therapies have transformed the treatment landscape of metastatic non-small cell lung cancer (mNSCLC) and are the preferred first-line (1L) treatment option for patients with mNSCLC harboring epidermal growth factor receptor (EGFR) exon 19 deletion (Ex19del) and exon 21 L858R substitution (L858R). However, recent real-world data on patient characteristics and treatment patterns in this population remain limited. This study aimed to describe treatment patterns, including immunotherapy use, in patients with EGFR-mutated (Ex19del/L858R) mNSCLC.

Methods

This retrospective study descriptively analyzed deidentified Integra Connect data for patients with documented EGFR-mutated (Ex19del/L858R) mNSCLC before initiating 1L treatment on or after January 1, 2018, with 1L initiation as the index date.

Results

Among 561 patients, the median age was 71 years; 369 (66.1% of 558 patients with known gender information) were female, 375 (66.8%) were White, and 290 (51.9% of 559 patients with known smoking status) never smoked. Ex19del was reported in 286 patients (51.0%) and L858R in 276 (49.2%). Most patients (n = 408, 72.7%) had documented next-generation sequencing testing. The mean (median) time from first recorded metastasis date to 1L initiation was 3.5 (1.1) months. The mean (median) time from first recorded EGFR-positive (Ex19del/L858R) test or test result and 1L initiation was 2.7 (0.5) months. The most common 1L treatments included osimertinib monotherapy (n = 419, 74.7%) and osimertinib combination therapies (n = 61, 10.9%). By data cutoff (June 30, 2024), 245 patients (43.7%) had received a second-line (2L) therapy. Common 2L treatments included chemotherapy-immunotherapy combinations (n = 48, 19.6%), osimertinib monotherapy (n = 42, 17.1%), and immunotherapy monotherapy (n = 25, 10.2%). Overall, 53 patients (9.4%) received immunotherapy as 1L treatment, while nearly one-third (n = 77, 31.4%) of those initiating 2L therapy received immunotherapy.

Conclusions

Despite guidelines recommending targeted therapy over immunotherapy in patients with EGFR-mutated (Ex19del/L858R) mNSCLC, immunotherapy use in 1L and 2L continues to be observed in the real world, highlighting substantial unmet needs and the need for more effective targeted treatments for these patients.

## Introduction

Lung cancer is the leading cause of cancer-related deaths in the United States (US), with an estimated 226,650 new cases and 124,730 deaths in 2025 [1]. Non-small cell lung cancer (NSCLC) accounts for 80% to 85% of all lung cancer cases [2]. Although the proportion of early-stage NSCLC at diagnosis has increased in recent years, partly because of the implementation of lung cancer screening programs, approximately 70% of newly diagnosed patients still present with advanced or metastatic disease [3-5]. Globally, about 12% of NSCLC cases carry epidermal growth factor receptor (EGFR) mutations [6], with exon 19 deletions (Ex19del) and exon 21 L858R point mutations (L858R) representing 85% to 90% of all EGFR mutations in NSCLC [7].

Targeted therapies have transformed the treatment paradigm for metastatic NSCLC (mNSCLC) with driver mutations. The current versions of the National Comprehensive Cancer Network (NCCN) guidelines (Version 4.2025) and the American Society of Clinical Oncology (ASCO) guidelines (Version 2024.3) recommend targeted therapies, alone or combined with chemotherapy, as first-line (1L) treatment options for patients with Ex19del and L858R mNSCLC [8,9]. Both guidelines list osimertinib, a third-generation EGFR tyrosine kinase inhibitor (TKI), as the preferred 1L therapy for these patients. Although the use of 1L osimertinib is associated with a median overall survival of approximately 39 months [10], nearly all patients eventually develop resistance [11].

Managing EGFR-mutated mNSCLC after progression on 1L osimertinib remains a challenge, and developing optimal treatment strategies is an area of active research [12-18]. However, recent real-world data on patient characteristics and treatment patterns in EGFR-mutated NSCLC, particularly regarding the patient journey across different care settings, remain limited. In this study, we aimed to describe patient characteristics and treatment patterns, including the use of immunotherapies, in patients with EGFR-mutated (Ex19del/L858R) mNSCLC.

A version of this information was previously presented as a poster at the 2025 ISPOR Conference in Montreal, Quebec, Canada, on May 13, 2025.

## Materials and methods

Study design and data source

This retrospective cohort study analyzed deidentified data in the Integra Connect database for patients with mNSCLC harboring Ex19del and/or L858R mutations who initiated treatment on or after January 1, 2018 [19]. These data were made available by Integra Connect, LLC, and used under license for the current study, and are not publicly available. Other researchers should contact Integra Connect (integraconnect.com). The Integra Connect database covers approximately 500 US care sites and includes data for about 3.2 million patients with at least two recorded clinical visits. It integrates longitudinal electronic health record data and testing data from a diverse network of community oncology practices, providing treatment-level information and enabling analysis of treatment patterns in real-world practice. In particular, it provides certain testing information that is not available in other databases, such as the Flatiron Enhanced Datamarts (EDMs) and SEER-Medicare data. This study was designed to describe real-world treatment patterns and did not have a prespecified hypothesis. This study does not constitute research involving human subjects and was therefore exempt from Institutional Review Board approval.

Study population

**Figure 1:**
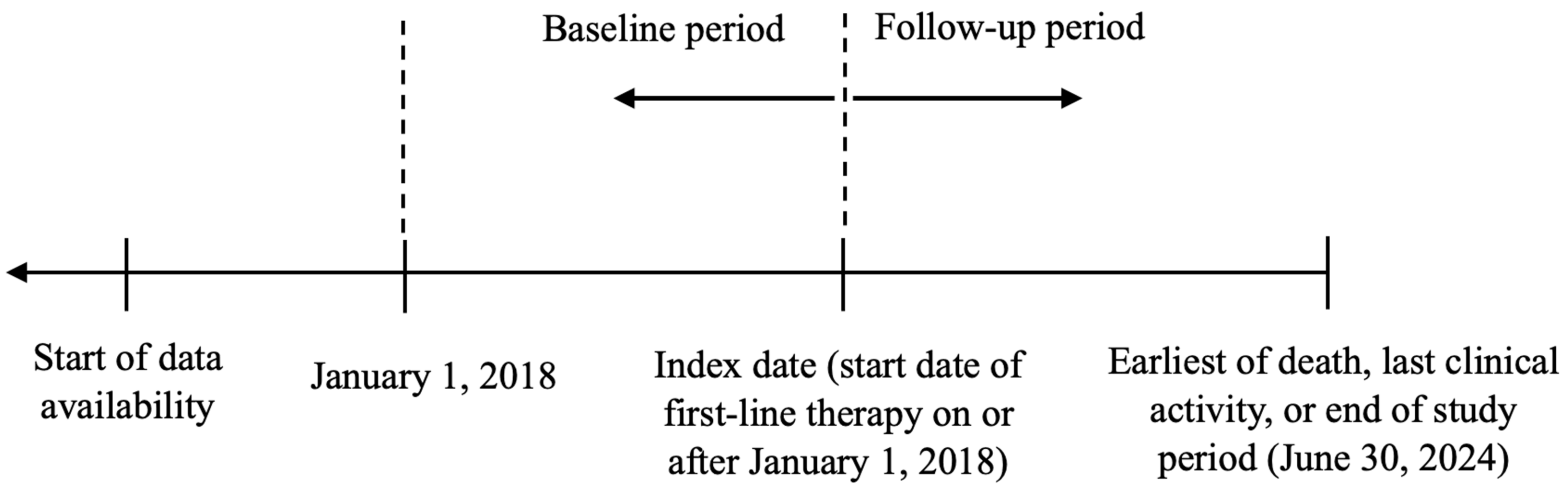
Study Design

Eligible patients had documented Ex19del/L858R mNSCLC prior to initiating the 1L treatment on or after January 1, 2018 (Figure 1, Appendix A). The start date of 1L therapy was defined as the index date. Patients were followed until the earliest of death, last clinical activity, or end of the study period (June 30, 2024). Patients were excluded if their 1L treatment was initiated before the recorded mNSCLC diagnosis date.

Outcomes and data analysis

Patient demographics and clinical characteristics (Appendix B) were assessed on or prior to the index date. Treatment patterns were described for 1L, second-line (2L), and third-line (3L) therapies. Lines of therapy (LOTs) were predefined by Integra Connect based on their business rules. A LOT includes the first identified drug and additional drugs administered within the following 60 days, and advances when a new drug is introduced or when there is a treatment gap of more than 365 days for oral drugs or more than 180 days for intravenous or subcutaneous injectables. Drug discontinuation alone and substitutions between carboplatin and cisplatin and between paclitaxel and nab-paclitaxel do not advance the LOT. Bevacizumab biosimilars can be used interchangeably with bevacizumab and do not advance the LOT. Treatments were classified into the following categories: osimertinib (alone or in combination with other therapies); first- or second-generation TKIs (alone or in combination with other therapies); chemotherapy (single-agent, doublet, or combined with anti-vascular endothelial growth factor (VEGF) agents or other chemotherapies); and immunotherapy-containing therapies. All variables and outcomes were descriptively summarized.

## Results

Patient characteristics

**Figure 2:**
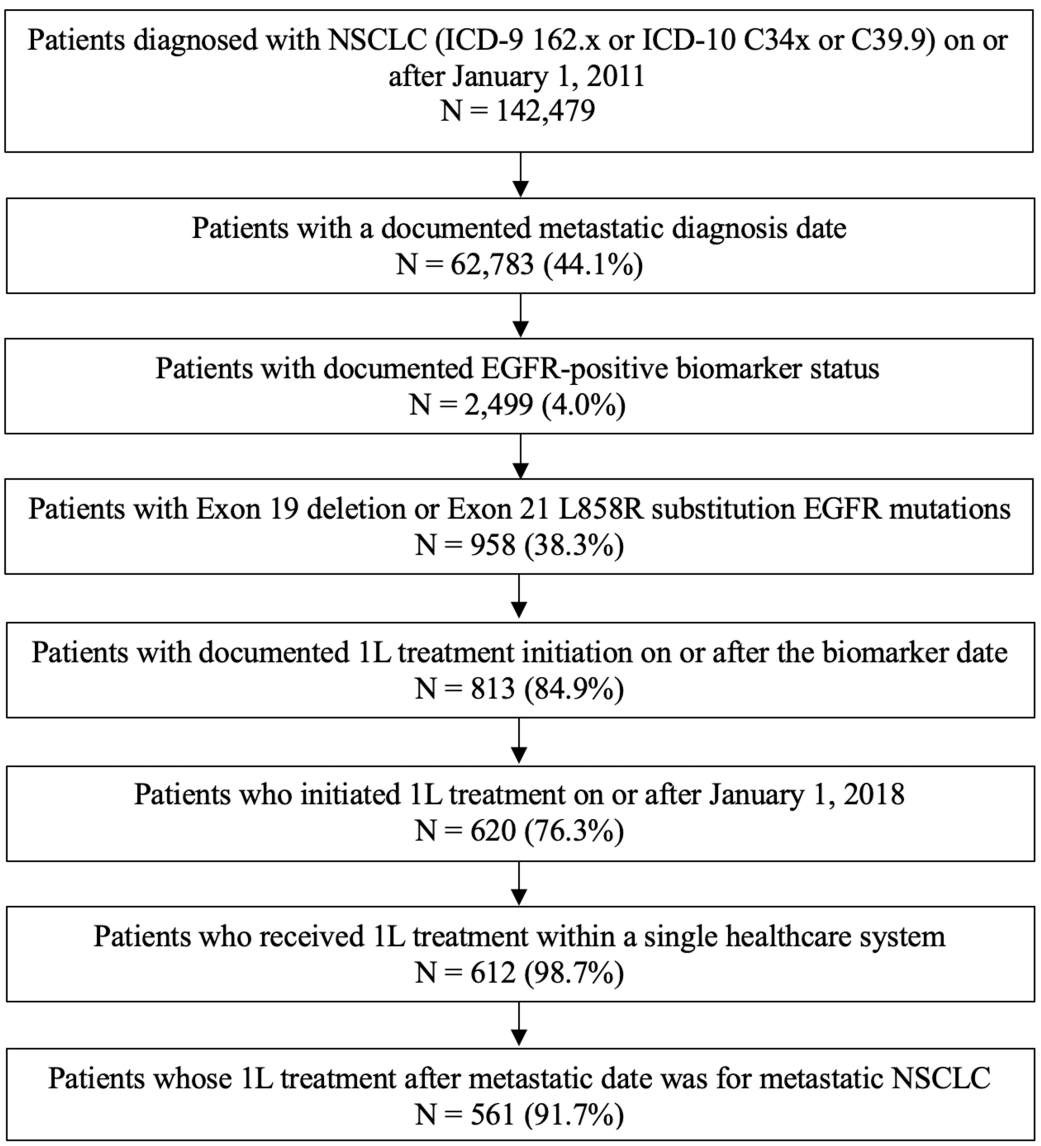
Patient Flowchart Note: Percentages were calculated using the sample size from the preceding step as the denominator. Abbreviations: 1L, first-line; EGFR, epidermal growth factor receptor; ICD, International Classification of Diseases; NSCLC, non-small cell lung cancer.

A total of 561 patients met the study criteria (median age 71 years; 66.1% female; 66.8% White; Figure 2, Table 1). Approximately half of the patients (51.9%) never smoked. The mean Charlson Comorbidity Index (CCI) score was 3.8 (2.4). The most common sites of metastasis were bone (43.0%), central nervous system (23.9%), and liver (12.5%). Ex19del was reported in 51.0% of patients, and the L858R mutation in 49.2%. Next-generation sequencing (NGS) testing was documented in 72.7% of patients. The mean (median) time from the first documented NSCLC metastasis date to 1L treatment initiation was 3.5 (1.1) months, and the mean (median) time from the first recorded EGFR-positive (Ex19del/L858R) test or test result date to 1L initiation was 2.7 (0.5) months.

**Table 1:** Patient Demographics and Baseline Clinical Characteristics (N = 561) ^a^Includes patients of other known or unknown races or ethnicities, as well as those with missing information. ^b^One patient had both Ex19del and L858R mutations.

Demographics	
Age, years	
Mean ± SD	70 ± 10
Median (IQR)	71 (64, 78)
Female, n (% of 558 patients with information available)	369 (66.1)
Current or former smokers, n (% of 559 patients with information available)	269 (48.1)
Patients who never smoked, n (% of 559 patients with information available)	290 (51.9)
Race, n (%)	
White	375 (66.8)
Black	64 (11.4)
Asian	48 (8.6)
Other^a^	74 (13.2)
Ethnicity, n (%)	
Hispanic or Latino	19 (3.4)
Non-Hispanic and non-Latino	393 (70.0)
Other^a^	149 (26.6)
Payer type, n (% of patients with information available)	
Commercial	110 (19.9)
Medicare or Medicaid	235 (42.5)
Other	207 (37.4)
Clinical characteristics	
CCI, mean ± SD	3.8 ± 2.4
Site of metastasis, n (%)	
Bone	241 (43.0)
CNS	134 (23.9)
Liver	70 (12.5)
Other	261 (46.5)
EGFR alteration, n (%)^b^	
Exon 19 deletion	286 (51.0)
L858R	276 (49.2)
NGS testing, n (%)	408 (72.7)
Time from the first documented metastasis date to the index date, months	
Mean ± SD	3.5 ± 20.5
Median (IQR)	1.1 (0.7, 1.8)
Time from the first documented EGFR-positive (Ex19del/L858R) test or result date to the index date, months	
Mean ± SD	2.7 ± 11.6
Median (IQR)	0.5 (0.3, 0.9)

Treatment patterns

**Figure 3:**
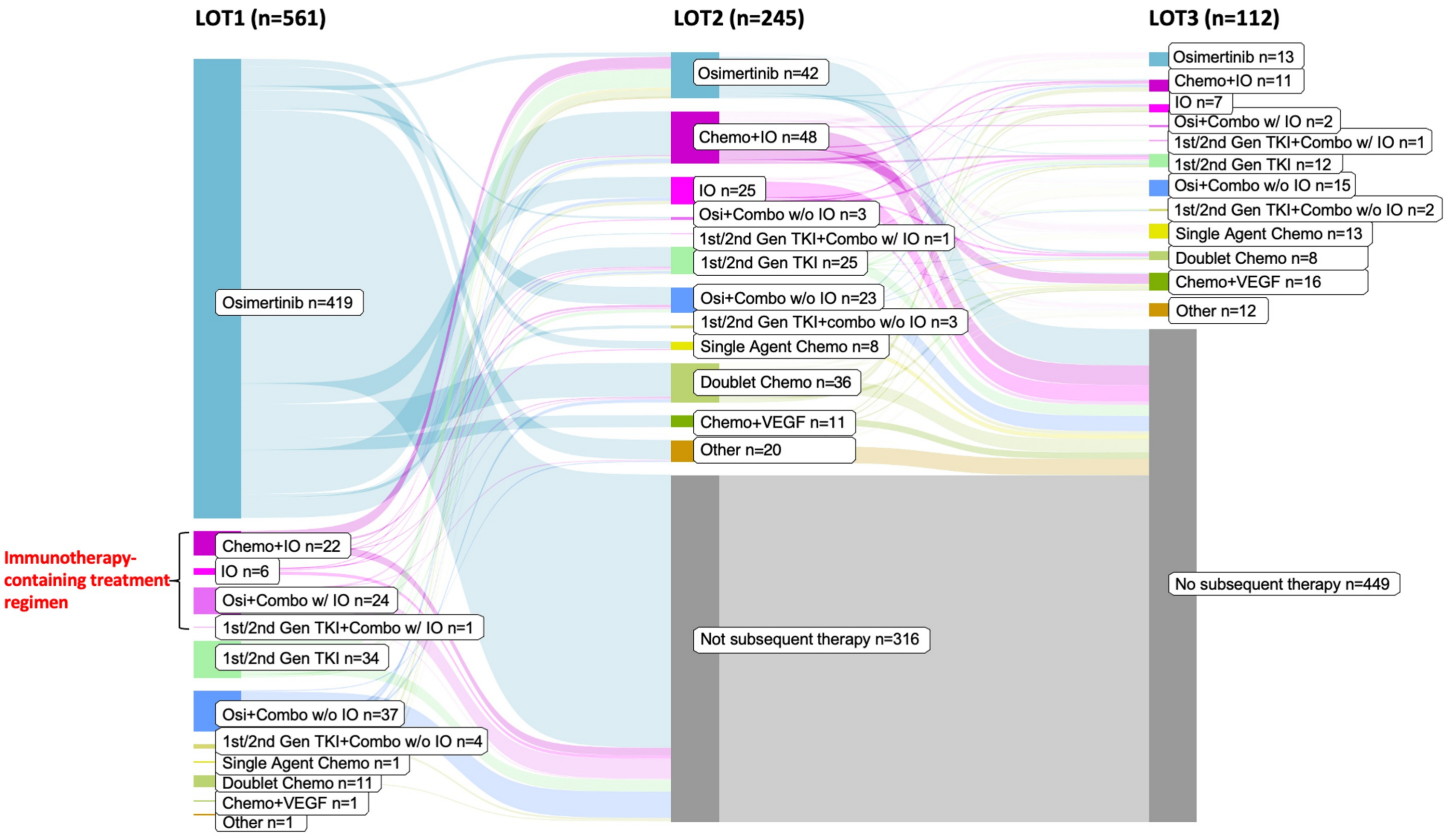
Treatment Patterns by Line of Therapy Abbreviations: Chemo, chemotherapy; IO, immunotherapy; LOT, line of therapy; TKI, tyrosine kinase inhibitor; VEGF, vascular endothelial growth factor.

The most common 1L treatments included osimertinib monotherapy (74.7%) and osimertinib-based combination therapies with or without immunotherapy (10.9%; Figure 3). The top five treatment regimens in the 1L setting included osimertinib monotherapy (74.7%), osimertinib combination without immunotherapy (6.6%), first- or second-generation TKIs (6.1%), osimertinib combination with immunotherapy (4.3%), and chemotherapy plus immunotherapy (3.9%). By the data cutoff date (June 30, 2024), 245 patients (43.7%) had received a 2L therapy. Among these patients, the most common 2L treatments included chemotherapy plus immunotherapy combinations (19.6%), osimertinib monotherapy (17.1%), doublet chemotherapy (14.7%), and immunotherapy (10.2%). Among patients who initiated 3L treatment by the data cutoff date (n = 111), the top five treatment regimens were chemotherapy plus a VEGF inhibitor (14.4%), osimertinib-based combinations without immunotherapy (12.6%), single-agent chemotherapy (12.6%), osimertinib monotherapy (11.7%), and first- or second-generation TKIs (10.8%) (Table 2).

**Table 2:** Most Commonly Used Regimen Types by Line of Therapy Abbreviations: 1L, first-line; 2L, second-line; 3L, third-line; TKI, tyrosine kinase inhibitor; VEGF, vascular endothelial growth factor.

Top five regimens	N (%)
1L	561
Osimertinib monotherapy	419 (74.7)
Osimertinib combination without immunotherapy	37 (6.6)
First or second generation TKI	34 (6.1)
Osimertinib combination with immunotherapy	24 (4.3)
Chemotherapy + immunotherapy	22 (3.9)
2L	245
Chemotherapy + immunotherapy	48 (19.6)
Osimertinib monotherapy	42 (17.1)
Doublet chemotherapy	36 (14.7)
Immunotherapy	25 (10.2)
First or second generation TKI	25 (10.2)
3L	111
Chemotherapy + VEGF	16 (14.4)
Osimertinib combination without immunotherapy	14 (12.6)
Single-agent chemotherapy	14 (12.6)
Osimertinib monotherapy	13 (11.7)
First or second generation TKI	12 (10.8)

Immunotherapy-containing regimens

Overall, 9.4% of patients received immunotherapy (as monotherapy or combination) as 1L treatment, while 31.4% of patients initiating 2L (n = 245) and 18.8% of those initiating 3L treatments (n = 111) received immunotherapy (Figure 3). The top immunotherapy-containing regimens were pembrolizumab plus carboplatin and pemetrexed with osimertinib (3.6%) or without osimertinib (2.9%) in 1L; pembrolizumab plus carboplatin and pemetrexed (13.1%), pembrolizumab (6.5%), and nivolumab (2.0%) in 2L among patients who initiated 2L treatment; and pembrolizumab (4.5%), pembrolizumab plus carboplatin and pemetrexed (2.7%), and pembrolizumab plus pemetrexed (1.8%) in 3L among those who received 3L treatment.

## Discussion

This retrospective analysis found that nearly 10% of patients with EGFR-mutated (Ex19del/L858R) mNSCLC received immunotherapy (either as monotherapy or in combination) as a 1L treatment, while nearly one-third of patients initiating 2L and one-fifth of those initiating 3L received immunotherapy-containing treatment regimens, despite the fact that current guidelines recommend targeted therapy over immunotherapy for EGFR-mutated mNSCLC [8,9]. Given that clinical trials have shown limited clinical benefit with immunotherapy (primarily immune checkpoint inhibitors (ICIs)) as monotherapy in EGFR-mutated NSCLC [12,13], and that combining ICIs with EGFR TKIs is associated with increased toxicity [14,15], these findings highlight a significant unmet need in this patient population. Consistent with findings from other real-world studies using different data sources [16,17], this study revealed a fragmented treatment landscape in 2L and later-line settings.

The overall treatment patterns observed in this study, including real-world immunotherapy use in EGFR-mutated NSCLC, broadly align with recent studies using different data sources [17,18]. In a population-based study using multicenter, nationwide electronic health record-derived data from the Flatiron database, Robinson et al. reported that among patients with advanced EGFR-mutated NSCLC between 2016 and 2022 whose disease progressed on 1L osimertinib (22.7%), 37% (197/538) received ICI-containing treatment in the 2L setting [17]. In another study also using the Flatiron database, Halmos et al. found that 23% of patients who received a platinum-based chemotherapy combination regimen between January 2011 and June 2020 after prior EGFR-TKI therapy received concomitant immunotherapy in the 2L setting [18]. Findings from this and other real-world studies suggest that treating EGFR-mutated mNSCLC remains challenging, with optimal treatment approaches, particularly following EGFR-TKI resistance, still under investigation. As new evidence emerges, the treatment paradigm and clinical guidelines for EGFR-mutated NSCLC are expected to evolve to guide informed decision-making and improve patient outcomes.

As with all real-world studies, this analysis has limitations related to the use of the available data, and the interpretation of the findings should be interpreted with caution. The US Integra Connect database primarily includes patients treated at community oncology practices participating in the Integra Connect network. The data were collected from different sites for purposes largely unknown to the authors. As a result, the findings from this study may be subject to selection bias and may not be generalizable to patients treated at academic or other non-participating practices. Similarly, the results from this study may not be easily reproduced using other external data. Additionally, medical services received outside of the participating practices may not be captured, resulting in incomplete treatment histories for some patients. However, it is unclear which patients have complete versus incomplete information regarding their disease history and treatment journey. Although data completeness appears to be higher among patients with curated data, the sampling strategy behind the data curation is unspecified, and there may be a risk of bias. Furthermore, certain important clinical variables, such as disease stage and the Eastern Cooperative Oncology Group (ECOG) performance status, are not consistently documented; biomarker and genomic testing data are not standardized across practices; certain key mutation data, such as STK11 and KEAP1, are not available, nor is PD-L1 information, which could help characterize the IO users; and death dates are not validated. Also, patients’ first documented metastasis date and first EGFR test date may be later than their actual first metastasis and EGFR test dates. Moreover, the database applied a 60-day drug identification window and allowed long treatment gaps for line advancement (e.g., 180 days for intravenous and subcutaneous medications and 365 days for oral drugs), which may lead to misclassification of LOT and suggest potential missingness in drug exposure data.

Despite these limitations, the Integra Connect database provides a large, nationally representative sample of cancer patients receiving treatment at community oncology practices. Its structured electronic health record data and longitudinal design enable timely analyses of real-world patient characteristics, biomarker testing, and treatment patterns across diverse patient populations. Using this database, the present analysis provides insights into the current treatment landscape for EGFR-mutated mNSCLC and highlights the challenges of managing the disease, especially in the second- and later-line settings. Further research using different data sources is warranted to characterize the patient journey.

## Conclusions

Despite guidelines recommending targeted therapy over immunotherapy for patients with mNSCLC harboring EGFR mutations (Ex19del/L858R), immunotherapy use in 1L and 2L settings continued to be commonly observed in the real world. Results from this study highlight substantial unmet needs and the need for more effective targeted treatment options for these patients. The conclusions should be interpreted in the context of the limitations and retrospective nature of the study.

## Appendices

Appendix A 

**Table 3:** Patient Inclusion and Exclusion Criteria Abbreviations: EGFR, epidermal growth factor receptor; LOT1, first line of therapy; NSCLC, non-small cell lung cancer.

Inclusion criteria
Patients who had metastatic information
Patients with EGFR mutation (i.e., Exon 19 deletion (Exon19) or Exon 21 L858R point mutation (Exon21 L858R))
Patients newly initiated on LOT1 treatment after metastatic NSCLC between January 1, 2018, and the latest dataset cut-off date
Exclusion criteria
Patients who progressed from localized lung cancer
Patients whose LOT1 initiation date was earlier than their mNSCLC diagnosis date

Appendix B 

**Table 4:** Study Variables and Measures Abbreviations: CCI, Charlson Comorbidity Index; CNS, central nervous system; LOT1, first line of therapy; NSCLC, non-small cell lung cancer; EGFR, epidermal growth factor receptor; TKI, tyrosine kinase inhibitor.

Demographics measured on or prior to the index date
Age
Gender
Smoking status
Race (Asian, Black or African American, White, other race, unknown/not documented)
Ethnicity (Hispanic or Latino, not Hispanic or Latino)
Year of index date
Time from metastatic NSCLC date to index date
Insurance type
Clinical characteristics measured on or prior to the index date
Comorbidities: Charlson Comorbidity Index (CCI) up to 12 months prior to index date (CCI score, individual CCI comorbidities)
Location of metastasis (CNS, liver, bone, other)
Treatment patterns
Treatment patterns were described for LOT1 and (subject to data availability) LOT2 and beyond, separately for the most common treatments, subject to data availability
Treatment type (e.g., third-generation TKI, first-second generation TKI, platinum-based chemotherapy, immuno-oncology therapy)
Biomarker testing
Biomarkers were measured on the test date (assessed subject to data availability and completeness) during the baseline period. Patients were classified into the following categories: EGFR Exon 19 deletion, EGFR Exon 21 L858R point mutation, TP53, T790M, MET, and other biomarkers/genetic factors of interest (subject to data availability)
Time between EGFR+ test result and initiation of LOT1 therapy
